# On the Employment of the Albuminate of Iron and Soda as a Therapeutic Agent

**Published:** 1853-09

**Authors:** Angellico Fabbri

**Affiliations:** Pharmaceutical Chemist at Gubbio


From the Bulletine della Scienze Mediche di Bologna, Nov. and Dec., 1852, p. 385. Dublin Quarterly Journal of Medical Science, May 1853.

*On the Employment of the Albuminate of Iron and Soda as a Therapeutic Agent, in various cases in which Ferruginous Preparations are indicated.* By ANGELLICO FABBRI, Pharmaceutical Chemist at Gubbio.

SIMPLE contact, at the ordinary temperature of the atmosphere, of white of egg with a salt of iron and soda, is capable of instantly producing a soluble albuminate of iron and soda, or an albuminferrate of the alkaline base. The chemical combination of this compound is such, that it is not altered by the yellow ferrocyanide of potassium, the most delicate test of the salts of iron, unless a few drops of acid: as for example the hydrochloric, be previously added to the soluble albuminate—thus proving that this decomposition cannot be effected by the agency of the alkalies, but only by some acids, since the potassium of the cyanide is not able to displace the oxide of iron, becoming oxidized at its expense, and setting the metal free, as occurs with the other ferruginous preparations. Considering that we find in the blood albumen, soda in excess, and iron, and having shown how these three bodies, by simple direct contact, form a soluble salt, the chemical combination of which is so powerful that it is not destroyed by the most delicate reagents, may we not fairly infer that the iron exists in the blood as an albuminate of iron and soda; and would it not therefore be reasonable to administer iron in the various diseases in which it is prescribed, principally in reference to the state of the sanguinous system, in the form of albuminate, as that in which nature itself has placed it within our organism—one of the products, so to speak, on which our life depends. When I read in works of chemistry that the yellow ferrocyanide of potassium is not capable of demonstrating the presence of iron in the blood, until a stream of chlorine has first been passed through the latter to destroy its coloring matter, I am confirmed in the opinion that the iron exists in that fluid as an albuminate of iron and soda, because this salt, requiring the addition of an acid to render it capable of detection by the cyanide, is supplihd with it by the chlorine, which, in destroying the organic coloring matter, becomes converted into hydrochloric acid by uniting with their hydrogen. Physicians have been long puzzled, and are still at a loss, how to administer iron, a most valuable remedy, in the manner most suitable to the internal organism; hence the great number of preparations of this metal. Some object to its saline combination with mineral acids, on the ground that these are inorganic, and they prefer giving it in the metalic or oxidised state, leaving to the acids of the stomach to form with it compounds which may be carried into the circulation. Others, unwilling to run the risk of having the greater part of the iron—little, or not at all acted upon—expelled with the fœces, prescribe in the saline state, but combined with organic vegetable acids; hence we have the malate, tannate, citrate, &c., of iron. Others, still more scrupulous, wish to have it united to acids of an animal nature, and prefer the lactate, the cyanide, &c.; and I, going still farther, would recommend its employment in the state of albuminate of iron and soda, requesting physicians to take into consideration what I have advanced, and to ascertain if practice will in this instance corroborate theory.

In preparing the albuminate of iron and soda, I employed the following process: Take 112 grs. of caustic soda, and 104 of sulphate of iron. Having dissolved both in a sufficient quantity of distilled water, let the solutions be poured on the whites of four eggs previously beaten up; let all now be shaken together and poured upon a filter to separate the hydrated oxide of iron which has precipitated, since all the iron is not in this case converted into albuminate. To the filtered liquid, which now contains, in addition to the albuminate, sulphate of soda formed by the decomposition of sulphate of iron by the soda present in excess, lime water is to be added, to decompose the sulphate of soda, by which an insoluble sulphate of lime is precipitated. To separate the latter, the mixture is to be again filtered; and as the filtered fluid will contain an excess of lime, it is to be subjected to the action of a stream of carbonic acid, care being taken to avoid using an excess of the latter, and again filtered to get rid of the insoluble carbonate of lime thus formed. The filtered fluid is now to be allowed to evaporate in a wide, shallow vessel, and with the aid of the heat of a stove, until it is reduced to a pint. A clear orange yellow, slightly saltish, chalybeate solution is thus obtained, which, as already mentioned, does not give a precipitate with ferrocyanide of potassium without the previous addition of an acid. Each ounce of this liquid contains approximatively four grains of the albuminate *plus* an excess of albumen and soda, as may be seen by referring to the process employed: the solution consequently has a slightly alkaline re-action. It is desirable that the soda should thus be present in excess, in order that the compound shall be conformable to the state in which it exists in the blood, where we find the albumen rendered alkaline by an excess of soda. This albuminate of iron and soda is represented by the following formula: $C^{30} H^{50} O^{10} + HO + Fe^2 O^3 + Na O + 2 HO = AL Fe^2 O^3 + 2 HO$ water. As the albumen loses a portion of its nitrogen in order to be converted into albuminic acid, we must suppose that a portion of the soda by its presence determines the formation of a fatty matter at the expence of other principles of the same albumen, and then becomes saponified. I have given the formula of the albuminate of iron and soda above, neglecting the excess of the albumen, which, though united to the liquid, perhaps with some other soluble salts of the albumen of the egg, (chlorides.) I do not consider to form a part of the saline compound, which may be obtained in radiated crystals by evaporating the solution to dryness.

